# Common Post-translational Modifications (PTMs) of Proteins: Analysis by Up-to-Date Analytical Techniques with an Emphasis on Barley

**DOI:** 10.1021/acs.jafc.3c00886

**Published:** 2023-10-04

**Authors:** Janette Bobalova, Dana Strouhalova, Pavel Bobal

**Affiliations:** † Institute of Analytical Chemistry of the CAS, v. v. i., Veveri 97, Brno 602 00, Czech Republic; ‡ Masaryk University, Department of Chemical Drugs, Faculty of Pharmacy, Palackeho 1946/1, Brno 612 00, Czech Republic

**Keywords:** barley, post-translational modification, protein, mass spectrometry

## Abstract

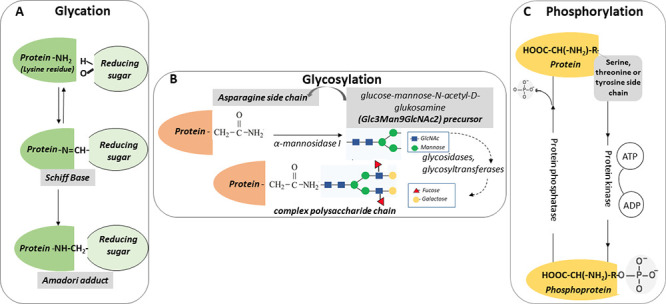

Post-translational modifications (PTMs) of biomacromolecules can be useful for understanding the processes by which a relatively small number of individual genes in a particular genome can generate enormous biological complexity in different organisms. The proteomes of barley and the brewing process were investigated by different techniques. However, their diverse and complex PTMs remain understudied. As standard analytical approaches have limitations, innovative analytical approaches need to be developed and applied in PTM studies. To make further progress in this field, it is necessary to specify the sites of modification, as well as to characterize individual isoforms with increased selectivity and sensitivity. This review summarizes advances in the PTM analysis of barley proteins, particularly those involving mass spectrometric detection. Our focus is on monitoring phosphorylation, glycation, and glycosylation, which critically influence functional behavior in metabolism and regulation in organisms.

## 1 Introduction

The introduction of a new structural element into the protein molecule after translation of the mRNA code allows the polypeptide chain to be transformed into different species with different functions. All living organisms must respond rapidly and efficiently to environmental changes through a tight regulatory system via molecular interactions of several hundred biomolecules.^1^ Due to multiple levels of regulation, such as PTMs and alternative splicing, a single gene is capable of producing several different proteins.^2^

Alternative uses of start and stop codons can give rise to different proteins. Proteins synthesized from these mRNAs may be differentially modified during or after translation. Hence, the same protein can be altered in many ways, leading to different variants. Since a cell is not a static entity, it is constantly responding to stimuli from both the external and internal environment.^3^ Therefore, many proteins are modified during or after synthesis in several ways, e.g., by cleavage of the polypeptide skeleton or by chemical modification of specific amino acid side chains. Post-translational modifications (PTMs serve a variety of purposes in different cellular processes, such as regulating enzymes, transducing signals, mediating subcellular localization of proteins, and interacting with proteins and different molecules.^4,5^

The PTMs can significantly affect protein localization and function. The identification and characterization of PTMs is therefore essential for understanding the regulation of metabolism, gene expression, and signaling in cells, and thus for the development of new therapeutics, as well as for the cultivation of high-quality plant varieties.

## 2 PTMs

Post-translational modifications can be categorized as follows: covalent incorporation of a chemical function to the side chain of a residue^6^ and cleavage of the peptide backbone.^7^ In the former case, a given protein may be post-translationally modified at many residues by the same group or the protein may undergo the addition of multiple types of covalently introduced groups. More than 400 different types of PTMs have been described to date.^8^ However, only some of these have been characterized in detail at the proteome level.

A wide range of different approaches has been used to study PTMs. A number of these approaches target individual proteins; for example, protein-specific phosphoantibodies are used to study cell signaling processes. More complex PTM analyses often depend on purification steps based on specific enrichment of modified proteins/peptides.^9^

### 2.1 Types of Protein PTMs

The most studied protein PTMs include glycosylation, phosphorylation, ubiquitination, acylation, methylation, nitration, and acetylation.^10^ The list of different types of PTMs is shown in Table 1. Some modifications, such as glycosylation, are usually permanent. Also, proteolytic cleavage represents the most prevalent irreversible PTM that all proteins undergo during their life cycle. In contrast, phosphorylation is reversible, can be used to regulate the activity of proteins in response to intracellular and extracellular signals, and is frequently involved in signaling pathways.

**Table 1:** List of Critical Post-translational Modifications^a^

modification	modification motif	amino acid modification site	number of publications (2017–2022)
acetylation	CH_3_CO	S, K	8 590
disulfide bond	S–S	C	4 580
glycation	(-Hex)_*n*_	K, R	5 331
glycosylation	(N-Glc-NAc)	N-X-S/T, X ≠ P	10 660
glutathionylation	GSH	cysteine thiol group	561
hydroxylation	(−OH)	side chain	1 458
lipidation	lipid/fatty acid	protein chain	631
methylation	(−CH_3_)	K	21 750
S-nitrosylation	(−NO)	cysteine thiol group	1 395
succinylation	CO–CH_2_–CH_2_–CO_2_H	K	584
SUMOylation	small ubiquitin-like modifier	protein chain	1 919
phosphorylation	HPO_3_	Y, S, T, H, D	68 208
ubiquitination	ubiquitin	K	9 505

a The number of published papers was obtained by entering the modification type in the Web of Science (October 2022).

#### 2.1.1 Glycosylation

There are two major types of glycosylation: *N*- and *O*-linked. The former involves glycans attached via amide nitrogen to asparagine.^11^ The latter reaction takes place at the hydroxy group of serine and threonine residues. Some glycoproteins contain sialic acid residues, whose ionizable nature significantly alters the behavior of the oligosaccharide moiety. It is well-known that glycosidic linkages in glycopeptides are more labile than peptide linkages and fragmentation of the glycan moiety predominates.^12^ Glycopeptides generate glycan-specific oxonium ions such as at *m*/*z* 204 (HexNAc), 163 (Hex), 292 (NeuNAc), and 366 (Hex-HexNAc) useful for structure assignment.^13^ The ion at *m*/*z* 204, as well as ions at *m*/*z* 186 and 168 arising from the elimination of water molecules, has been shown to be indicative for both *N*-glycans and *O*-glycans.^14^ Also, mass spectra data sets can be validated for constant neutral loss of terminal monosaccharides.^15^

The analysis of PTMs of proteins mainly requires their selective enrichment due to the low stoichiometry of modifications relative to nonmodified proteins, low abundance, heterogeneity of derivatives, and the majority of interfering nonmodified proteins. For enrichment of glycopeptides, methods based on lectin chromatography,^16^ hydrophilic interaction chromatography (HILIC),^17^ reverse phase chromatography, electrostatic repulsion hydrophilic interaction chromatography (ERLIC),^18^ cation-exchange chromatography,^19^ boronic acid-functionalized particles,^20^ and hydrazine chemistry were used.^21^ Affinity chromatography with lectins shows the changes in glycosylation patterns;^16^ it possesses a high specificity for the particular type of glycan moiety.

Cellulose, amide, and zwitterionic phases were used for HILIC separation. It can enrich hydrophilic non-glycosylated peptides as impurities.^22^ Under reversed-phase LC conditions, glycopeptides have been enriched using porous graphite.^19^ Glycopeptide resolution in ERLIC is based on the structure of glycans; it exhibits potential use for separating isomers caused by carbohydrate microheterogeneity. The principle predeterminate use of this method is for the enrichment of sialoglycopeptides.^18^ Glycopeptides containing sialic acid can be retained very efficiently on TiO_2_ under conditions in which unmodified peptides or neutral glycopeptides do not bind.^23^ Contamination with phosphorylated peptides is suppressed by phosphatase treatment, whereas the presence of sulfated peptides is not ruled out.^24^ Boronate affinity chromatography (BAC) is characterized by formation of covalent bonds between immobilized boronic acid and a cis arrangement of vicinal hydroxy groups. Gold nanoparticles functionalized with phenylboronic acid were utilized for the on-plate specific selective concentration of glycosylated peptides before matrix-assisted laser desorption/ionization (MALDI) analysis. However, BAC is unique not only for glycopeptides but for all compounds containing cis-diol moieties.^25^

The extraction selectivity can be improved by the conjunction of independent separation principles. Zwitterionic HILIC and RP-18 chromatography is orthogonal and complementary for the separation of glycopeptides. While HILIC distinguishes the glycan group, RP-18 facilitates separation by peptide sequence and degree of sialylation.

##### 2.1.1.1 Formation of N-Glycoproteins

In plants, the most widely studied protein modification is *N*-glycosylation. The oligosaccharide precursor Glc3Man9GlcNAc2 (glucose-mannose-*N*-acetyl-d-glucosamine) first attaches to the Asn residue, and the formed glycoprotein is then moved from the ER via the Golgi apparatus to its destination. During this process, *N*-glycan undergoes several changes consisting of the final removal and addition of sugar groups by different glycosidases and glycosyltransferases.^26^ A potential N-X-S/T glycoprotein sequence may be present multiple times in a polypeptide chain, but not all potential *N*-glycosylation sites are necessarily occupied.^27^

Plant *N*-glycans differ from their animal counterparts in several properties.^28^ As an example, plants do not produce and activate sialic acid and are unable to perform β(1,4)-galactosylation. Several attempts have been made to achieve this. The formation of stable protein sialylation in *Physcomitrella* has been described by Bohlender et al.^29^

#### 2.1.2 Nonenzymatic Glycosylation (Glycation)

**Figure 1:**
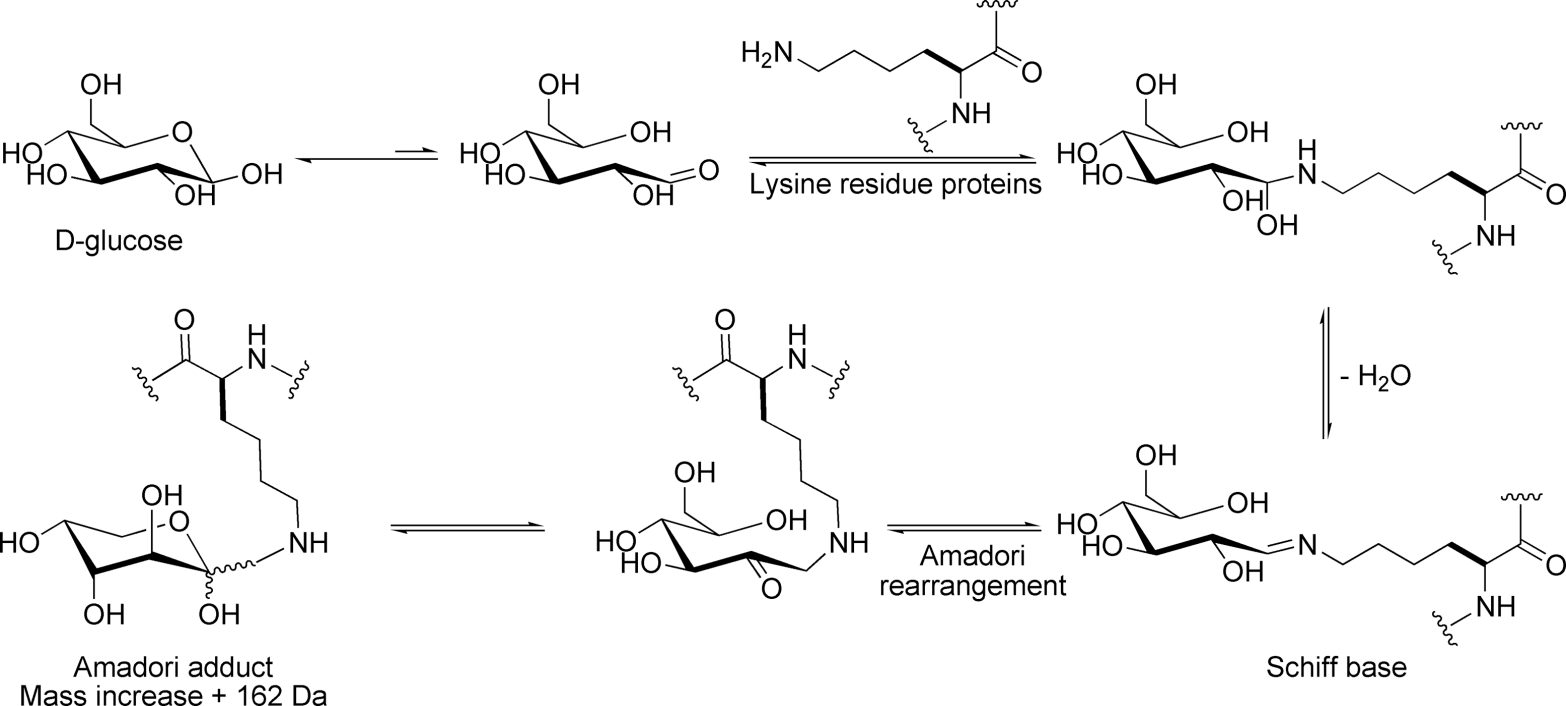
Scheme of nonenzymatic glycation.

Proteins and peptides are very often modified by nonenzymatic glycation. This reaction is also called the Maillard reaction, whose chemistry is highlighted in Figure 1.^30^ The free amine groups of lysine and guanidine groups of arginine in peptides and proteins react with reducing carbohydrates, α-oxoaldehydes, and their derivatives to form Schiff bases, which are not stable and are rapidly rearranged to the so-called Amadori products.

#### 2.1.3 Phosphorylation

Phosphorylation usually occurs on serine, threonine, and tyrosine residues. Phosphorylation is reversible and controlled by the action of kinases and phosphatases. Another important acidic moiety introduced during PTM is the SO_3_H group, which is formed by oxidation of the sulfanyl group of cysteine or transferred to hydroxyls of tyrosine, serine, or threonine in proteins.^31^

Identification of phosphopeptides is deduced from the neutral loss of 98 (Ser, Thr) or 80 (Ser, Thr, Tyr) Da in positive-ion mode and, respectively, 79 or 63 (Ser, Thr, Tyr) in negative-ion mode. This procedure, combined with the phosphatase treatment, leads to a mass shift of 80 Da.^32^ Unlike the phosphorylated peptides, which retained the moiety at low-energy collision, isobaric sulfated peptides lost SO_3_ (80 Da) before backbone fragmentation occurred.^33^ Moreover, the instruments can be capable of measuring weight with high accuracy to distinguish phosphate versus a sulfate group based on their mass difference (9.5 mDa).^34^

Isolation of phosphopeptides involves immunoprecipitation with phosphospecific antibodies,^35,36^ immobilized metal affinity chromatography (IMAC),^32^ metal oxide affinity chromatography (MOAC), HILIC, ERLIC, and strong anion exchange chromatography (SAX).^37^

A technique based on cation exchange separation has been developed for the enrichment of Tyr sulfated peptides. Sulfation sites are characterized by the presence of several acidic amino acids in their vicinity.^38^ For the detection, purification, and monitoring of sulfonation of tyrosine-sulfated proteins, monoclonal antibodies have been produced.^39^

Several described isolation approaches show broad specificity, and therefore, they can potentially simultaneously enrich various post-translationally modified peptides. Multistep parallel screening of glycosylation and phosphorylation was suggested by Zhao et al.^40^ Some results demonstrating simultaneous purification of neutral glycopeptides and phosphopeptides on porous titania microspheres have been published.

## 3 Protein PTMs and Innovative Analytical Techniques

Traditionally, PTMs have been identified by Edman degradation,^41^ radioactive isotope labeling,^42^ and Western blot immunoanalysis.^43^

Using Edman cleavage, the amino acid sequence of a given protein or peptide can be determined. The method allows the peptide to be labeled and cleaved from the *N*-terminus without breaking the peptide bonds between the other amino acid residues. Unfortunately, this method is not suitable for the analysis of shorter sequences.^44^ In 2020, Kristoffersen used Edman degradation sequencing and immunological detection to separate and characterize essential barley seed proteins.^45^ Their work identified three hordeins and detected a fourth hordein, while other partially sequenced proteins appear to have roles in plant stress or defense.

Due to its high sensitivity, mass spectrometry is a versatile tool for locating and mapping PTM sites. MS-based strategies typically involve several steps: enzymatic digestion of the protein, concentration of modified peptides, peptide sequencing by MS/MS, and statistical evaluation of results using databases. MALDI, electrospray ionization (ESI) mass spectrometry, and surface-enhanced laser desorption/ionization MS are frequently employed for this purpose.^46^ The shotgun sequencing approach is based on the parallel action of multiple proteases with different specificities with analysis by the HPLC-MS/MS technique. It is useful to convert PTMs that are labile during MS(/MS) experiments to stable species, preferably by introduced tagging.^47^

**Figure 2:**
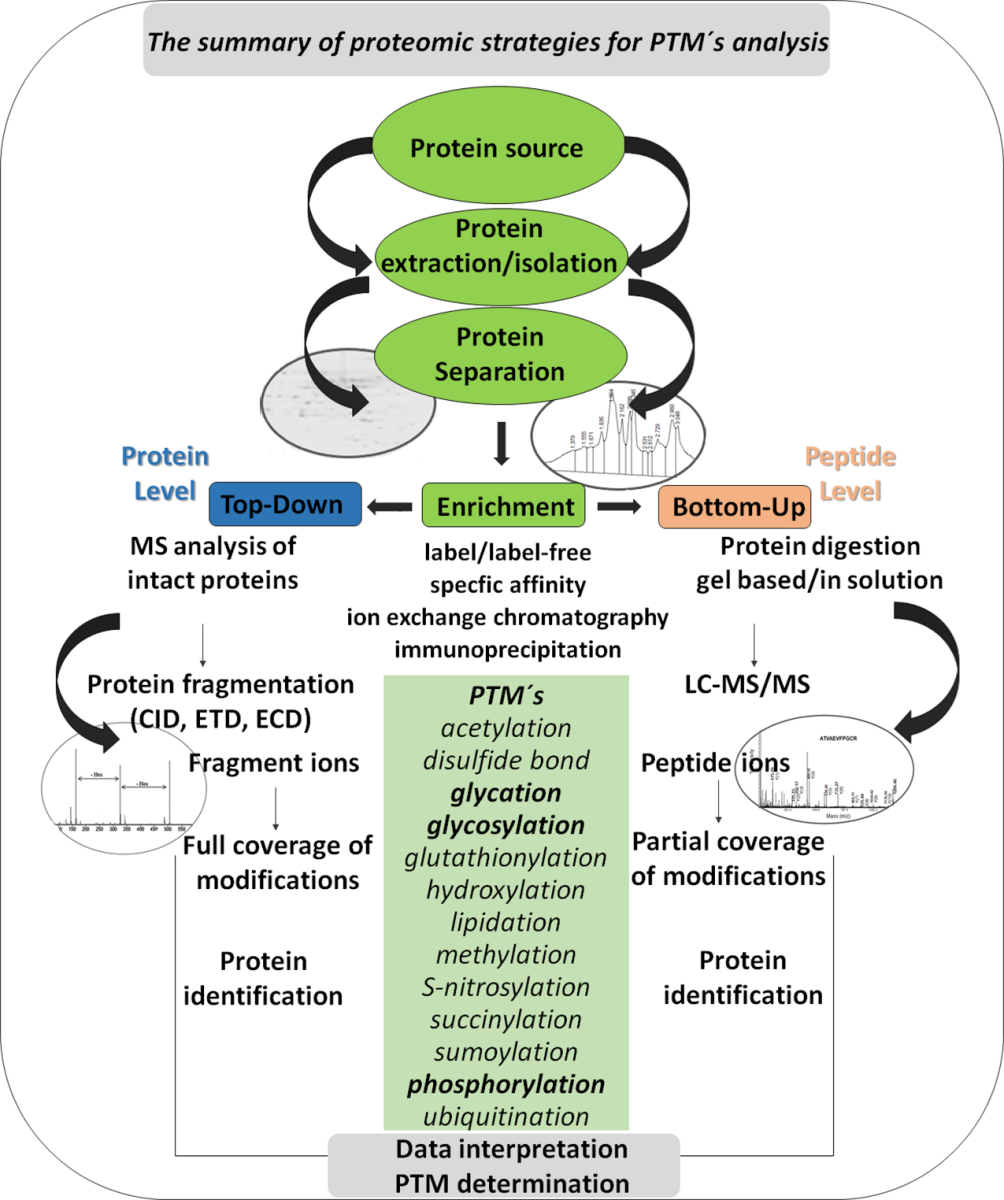
Scheme of MS-based proteomic strategies for PTM analysis.

Collision induced dissociation (CID) and electron capture/transfer dissociation (ECD/ETD) usually give complementary information on glycopeptides. In CID, fragmentation of protonated glycopeptides occurs mainly at glycosidic linkages, producing carbohydrate oxonium ions and/or fragment ions resulting from the repeated loss of monosaccharide units from both ends of the glycan.^48^ The schematic representation of proteomic MS-based strategies commonly used for PTM analysis is depicted in Figure 2.

It is well-known that an appropriate matrix selection for MS measurements often determines the outcome. The binary matrices 2,5-dihydroxybenzoic acid (2,5-DHB)/α-cyano-4-hydroxycinnamic acid (CHCA) and 2,5-DHB/sinapinic acid (SA) can also be used for MALDI-MS analysis of glycoproteins and determination of other modified proteins.^49^

Capillary electrophoresis (CE) also offers a simple application for glycan analysis. Since most glycans and oligosaccharides generally do not contain a chromophore or fluorophore in their natural structure and are difficult to ionize, it is necessary to perform a labeling step prior to their analysis using MS. Recently, several new labels and labeling strategies have emerged for carbohydrate analysis using CE. Labeling of carbohydrate molecules with a carbonyl group can be achieved by several reactions. It should be mentioned that the most common chemical labeling method is the reductive amination reaction, which is used to derivatize carbohydrates. At present, labeling with 8-aminopyrene-1,3,6-trisulfonic acid trisodium salt (APTS) is mainly used.^50^

Classical approaches have limitations such as a gap between shotgun-based approaches and clinically relevant results. Therefore, new approaches, sample preparations, and instrument innovations need to be introduced. This includes the involvement of, e.g., microchip electromigration methods, advanced electromigration methods with MS detection, and a combination of nanotechnology, surface enzyme reactions, and microfluidic networks.^51,52^

**Figure 3:**
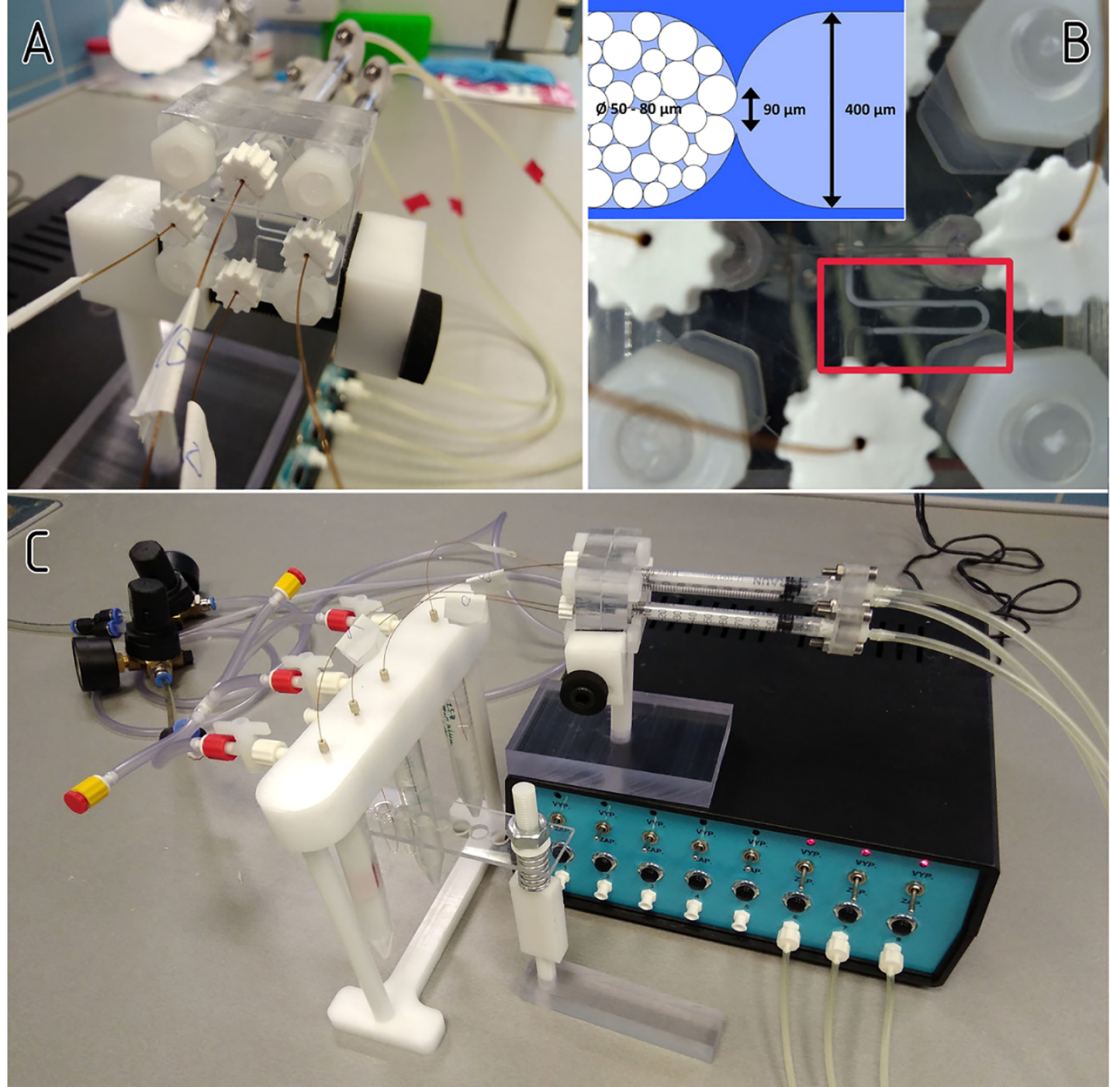
(A) Assembled microfluidic device. (B) UltraLink microbeads packed into the device. (C) The device is connected to a constantly pressurized fluid dispenser. Which fluid is injected is determined by the valves and CDA-manifold. Reprinted with permission from ref (53). Copyright 2019 John Wiley and Sons.

The use of microfluidics in analyzing glycoproteins and glycans has become prevalent. One of the many systems researchers choose is the 1200 Infinity Series HPLC-Chip/MS instrument, which features a ChipCube interface for ESI. The columns, injection channel, ESI tip, and connections are housed on a laminated polyimide chip, which is inserted into the ChipCube interface in a credit-card-sized stainless-steel holder, with the Infinity LC instrument serving as the solvent delivery system. In 2019 Novotný et al.^53^ introduced a microfluidic device for isolation and purification of glycoprotein samples. As seen in Figure 3, the microfluidic chip contained a bed of microbeads coupled to immobilized biomolecules that served as a bioaffinity column. The dosing of the different fluids required for each step of the sample treatment procedure was controlled by integrated pneumatic valves. The microfluidic device was combined with the label-free voltammetric analysis using catalytic peak H, which also represents an alternative method for protein detection. The advantage of using peak H is that all proteins mainly produce this intrinsic signal.

Combinatorial peptide ligand libraries (CPLLs) designed for low-abundance proteins are also used in proteomics. Eliminating high-abundance proteins is very efficient, but detecting low-abundance proteins remains limited due to their dilute presence. What seems interesting about reducing the number of high abundance proteins while increasing the number of low abundance proteins is the possibility of detecting more proteins and their PTMs.^54^ CPLL technology is characterized by three important principles: (1) a mixed set of affinity resins covering the largest spectrum of affinity ligands to statistically provide a partner ligand for each protein present in the samples, (2) the saturation effect of the capture due to a large number of sample overload, and (3) the exhaustiveness of protein recovery for full analysis. Although CPLL technology is currently used to track low abundance proteins, some research groups are still comparing the immunodepletion method with enrichment methods to obtain the best results.^54^

Data-dependent acquisition (DDA) and data-independent acquisition (DIA) methods along with sequential window acquisition of all theoretical mass spectra (SWATH-MS) are also more widely used for protein identification and quantification.^55^

iTRAQ, an advanced MS-based procedure, is also used for the relative and absolute quantification of proteins and their modification by the derivatization of primary NH_2_ groups in intact proteins. All peptides in a sample are labeled with an isobaric tag (a reporter group), individual samples with different ones. During the MS/MS experiments, the reporter groups produce different ions for a particular example (e.g., *m*/*z* 114 and 117). The ratios of intensities of the different reporter ions provide the relative abundances of protein in compared samples.^56^ iTRAQ is mainly used to analyze different cellular systems and to describe changes in protein expression related to cancer; it is also applied in other areas of agricultural research.^57,58^

To identify changes in the electrophoretic mobility of phosphoproteins, Okawara et al. developed the Phos-tag SDS-PAGE method. However, there was a problem with this technique; when SDS-PAGE and Phos-tag SDS-PAGE were used separately, detecting mobility shifts using Mn^2+^ Phos-tag was often challenging. The authors solved this problem by diagonal electrophoresis with Phos-tag, in which SDS-PAGE and Phos-tag SDS-PAGE patterns are presented on a single gel.^59^

Inorganic nanofibrous materials based on titanium dioxide or zirconia have been developed for the selective and efficient enrichment of phosphopeptides for MALDI/MS detection. Compared to conventional materials, the present nanofibrous materials exhibit much higher permeability, allowing their use in column packing and pipet tip format without the need for high pressure.^60^

## 4 Post-translational Modifications of Barley Proteins and Different Approaches to Their Determination

Barley (*Hordeum vulgare*) is considered to be one of the first cereals grown for human consumption. It is known to have been the food of gladiators and soldiers. However, barley is grown worldwide for a number of reasons, mainly as a feed grain and also for malting purposes. Barley is used in the production of beer and whisky and is also used in small quantities as a coffee substitute. Two types of botanical barley are distinguished: two-row and six-row genotypes. Six-row barley generally has a higher protein concentration in the grain than two-row barley, but both types are suitable for malting after modification of the cultivation method.

Two-row malting barley is traditionally grown in Europe, Australia, and South America, while six-row malting barley is more frequently grown in North America.^61^

Barley grain consists of a mixture of carbohydrates, proteins, lipids, minerals, and other important substances.^62,63^ Although protein accounts for approximately 10% of dry matter; it has been studied in detail. Their quantity and composition influence the suitability and quality of the barley grain for the final use, with the finished beer containing approximately one-third barley protein.^64^ Many proteins are modified during the malting process. In this review, we selected the most crucial barley proteins, their chosen modification, and analytical strategies used for their identification over the past few years (Table 2).

**Table 2:** Overview of the Essential Glycosylated, Glycated, and Phosphorylated Barley Proteins and Used Analytical Approaches for Their Study

PTM	protein	analytical approach	ref
glycosylation	α-amylase inhibitor BDAI-1, α-amylase inhibitor BMAI-1, α-amylase/trypsin inhibitor CM16, β-amylase, β-glucosidase, aldose reductase	HPLC (monolithic lectin affinity column), MALDI MS/MS	(65)
	β-d-xylosidase, peroxidase 1, α-amylase inhibitor BMAI-1, α-mannosidase, purple acid phosphatase isoform a	ConA chromatography, SDS-PAGE, MALDI MS/MS	(66)
	amylase/trypsin inhibitor family, pUP38, barwin	SDS-PAGE, MALDI-MS/MS, iTRAQ quantification	(57)
	β-amylase, β-glucosidase	HPLC-MALDI-MS/MS, iTRAQ quantification	(58)
	chaperonin HSP60, basic pathogenesis-related protein PR5	2D DIGE, LC-MS	(67)
	γ-hordein-3, D-hordein, putative γ2-hordein, C-hordein	LC-ESI-MS/MS, DIA-MS, DDA-MS	(68)
	peroxidase 1	2-DE-MALDI-TOF MS off-line Q-TOF MS/MS	(69)
	basic pathogenesis-related protein PR5	LC-MS/MS, MALDI-TOF-MS	(70)
	peroxidase 1, serpin-Z7, α-amylase inhibitor BDAI-1, serine carboxypeptidase II, ribose-5-phosphate isomerase 2, β-tubulin, α-amylase/trypsin inhibitor CM16, germin-like protein, glutamate receptor	DIA/SWATH-MS, LC-MS/MS	(11)
	serpin-Z4, serpin-Z7, α-amylase inhibitor BMAI-1, germin-like protein, barperm 1, malate dehydrogenase	DDA LC-MS/MS	(71)
glycation	protein Z4, protein Z7	2-DE SDS-PAGE, immunoblotting	(72)
	ns-LPT1, protein Z	LC-MS	(73)
	ns-LPT1	2-DE-MS	(74)
	ns-LPT1, ns-LPT2, protein Z	2D-HPLC- MALDI-TOF/TOF	(75)
	ns-LPT1, protein Z4	Nanoflow-HPLC-ESI-MS/MS	(76)
	ns-LPT1, protein Z	DF-IEF, MALDI-MS (linear)	(77)
	ns-LPT1, ns-LPT2, protein Z	MALDI-MS (linear)	(78, 79)
	ns-LPT1, ns-LPT2, protein Z	top-down MALDI/MS	(80)
	protein Z	bottom-up MALDI-MS/MS	(81)
	ns-LPT1, ns-LPT2, protein Z4, protein Z7	2-DE, MALDI-TOF/TOF MS	(69)
	β-amylase, α-amylase/trypsin inhibitor CMd, α-amylase/trypsin inhibitor CMa, cold regulated protein, protease inhibitor 13, endochitinase 2	2D-LC-MS/MS	(82)
	B1-hordein, γ-hordein-3, B3-hordein, putative γ2-hordein, serpin-Z4, thionin BTH7, 26 kDa endochitinase 2, dehydrin DHN1, cold-regulated protein	LC-ESI-MS/MS, DIA-MS, DDA-MS	(68)
	serpin-Z4, serpin-Z7, α-amylase/trypsin inhibitor CMd, α-amylase inhibitor BDAI-1, ns-LPT1, ns-LPT2, ubiquitin-like protein, cold-regulated protein	DIA/SWATH-MS, LC-MS/MS	(11)
phosphorylation	glycine-rich RNA-binding protein, triosephosphate isomerase, glyceraldehyde-3-phosphate dehydrogenase	MO affinity chromatography, LC-MS/MS	(83)
	chaperonin HSP60, heat shock protein 70, glycine-rich RNA-binding protein	2D DIGE, LC-MS	(67)
	calcium-dependent protein kinase 2	LC-MS/MS, nano-UPLC RP-C18 column	(84, 85)
	inorganic pyrophosphatase, hexose transporter HvSTP, sodium/proton exchanger HvNHX1, potassium transporter HAK2	IMAC/TiO2 affinity chromatography LC-ESI-MS/MS	(86)
	nucleoside diphosphate kinase, glutathione-S-transferase, calmodulin, β-tubulin, 14-3-3-like protein A, calcium-dependent protein kinase 2	co-immunoprecipitation (co-IP) assay MS/MS	(87)
	nucleoside diphosphate kinase, calmodulin, 2-Cys peroxiredoxin BAS1, glycine-rich RNA-binding protein, calcium-dependent protein kinase 2, triosephosphate isomerase, chaperonin HSP60, inorganic pyrophosphatase, glutamate receptor, purple acid phosphatase isoform a, ribose-5-phosphate isomerase 2, malate dehydrogenase	LC-MS/MS immune-affinity antibody analysis	(88)

### 4.1 Glycosylation

The well-known proteomics approaches include MALDI-TOF, SDS-PAGE, 2D differential gel electrophoresis (DIGE), MS, two-dimensional gel electrophoresis (2DE), LC-MS/MS LC-ESI-MS/MS, iTRAQ, and peptide mass fingerprinting (PMF) have been applied in barley to determine PTMs.^57,65−70^ Several published results confirmed that a combination of multiple omics approaches could be beneficial in identifying potential candidate genes, their pathways, and the covalent modification of proteins. Generally, lectin affinity chromatography in various combinations with gel electrophoresis, liquid chromatography (LC), and MS techniques is the most widespread approach for glycoprotein analysis.^65,66^ In quantitative proteomics, the iTRAQ method showed differential expression of selected barley allergenic proteins (e.g., pUP38, barwin) during food processing.^57^

The methods used in proteomics are also applicable to glycoproteomics. Nevertheless, the natural heterogeneity and dynamic nature of glycosylation make it difficult to identify and quantify. For this reason, specialized software is needed to identify the glycan component attached to peptides or proteins. The obtained MS/MS spectra of these species are more complex, because of the fragmentation of the glycans. In general, glycosylated species are usually less abundant than peptides due to glycan heterogeneity and reduced ionization efficiency during MS. Both labeled and unlabeled approaches are widely used to quantify glycosylation by MS, including DDA, DIA, and multiple reaction monitoring.^11,71^ Kerr et al.^68^ recently used DIA/SWATH-MS and reported very high PTM diversity in 23 commercial beers, focusing mainly on proteolysis and glycosylation. As shown in Table 2, the main group of high-mannose *N*-glycosylated proteins was represented by some well-known barley-pathogenesis-related proteins, such as protease/α-amylase inhibitors, germin-like proteins, peroxidase, and hordeins.

### 4.2 Glycation

Glycation is the second most common modification of barley proteins (apart from proteolysis or protein degradation). Approximately 3 to 7% of the glycated proteins in beer may be responsible for the formation of haze and approximately 25% for the stability of foam. Among the components responsible for the formation of haze is a proline-rich peptide derived from hordeins ranging in size from 15 to 32 kDa. The carbohydrate components in this case consist mainly of hexose. The major identified proteins from this group were barley lipid transfer protein (LTP) and Z protein (Table 2).^72−81,69^

Malt varieties differed in the extent of glycation of LTP1 and protein Z fragments. This was reflected by ladders of MS peaks differing in mass by approximately 162 Da. Bobálová et al.^81^ showed that some protein glycation occurred on the second day of malting. An efficient method for rapid identification of malt proteins and determination of PTMs, particularly glycation, is the application of monolithic chromatographic media and analysis of intact proteins by MALDI-TOF MS. Fractionation of aqueous barley extract on monolithic convective interaction media (CIM) disks with diethylamine (DEAE) column yielded better MS spectra and allowed rapid detection of technologically induced glycation of the protein fragment Z (*m*/*z* 4.0 kDa), LTP1 (*m*/*z* 7.5 kDa) and LTP2.^79^

As shown in Table 2, previous studies have characterized glycated proteins by gel electrophoresis and mass spectrometry. Some authors have also used a combination of LC and mass spectrometry to examine protein structure.^73,76^ Cho et al.^82^ used 2D-LC-MS/MS to enrich and analyze glycated proteins. Also, linear mode MALDI-MS can be used to monitor the malting process by characterizing the glycation of LTP proteins and protein Z, which is a fast and inexpensive analysis.^78^

Isoelectric focusing (IEF) devices appear to be an interesting alternative to LC. Their advantages are that they are significantly more straightforward, are cheaper than LC, and are faster than PAGE. The method can also be used to study PTMs without special chemical requirements, sample preparation, and purification procedures. Mazanec et al. applied this approach for the study of nonenzymatic glycosylation of major barley proteins LTP and protein Z.^77^

### 4.3 Phosphorylation

Most of the structural and metabolic proteins summarized in Table 2 belong to the phosphoprotein family. Within the previously mentioned mass spectrometry-based approaches, a wide range of enrichment techniques is used to capture phosphoproteins/phosphopeptides.^83−87,67^ Recently, a phosphoproteomic study of barley grain based on IMAC or TiO_2_ affinity chromatography followed by LC-MS/MS analysis was used to compare phosphoprotein regulation during imbibition. Phosphosignaling networks in barley grains were examined using large-scale phosphopeptide analysis to explore potential changes in ripening response pathways.^83^

Generally, study of phosphoprotein profiles indicates the major influence of protein phosphorylation in seed germination and can serve for monitoring of physiological changes occurring during germination and dormancy, as well as stress conditions.^84,85^

In recent years, immunoaffinity strategies have been widely used for the enrichment of phosphoproteins involved in various biological processes and metabolic pathways, such as Ca^2+^ signaling pathways, carbohydrate metabolism, or signaling pathways.^87,88^ In addition, Wang et al.^87^ performed a co-immunoprecipitation (co-IP) assay (using phospho-serine) and MS/MS analysis for specific KEGG pathway enrichment. The results show that phosphoproteins, found mainly in the nucleus and chloroplast, play a key role in processes of the MAPK signaling pathway such as RNA transport, endocytosis, or RNA degradation.

Finally, a brief overview of the essential proteins and their PTMs identified in barley is given in Table 3. We focused on the three most important groups of proteins classified according to their biological functions: (a) storage proteins (prolamins/hordeins); (b) structural and metabolic proteins; and (c) pathogenesis-related proteins. Structural and metabolic proteins, mainly kinases, transferases, and glycoside hydrolases, represent the main proteins studied in this review. As expected, protein glycosylation occurs predominantly in all three types of proteins: storage proteins, pathogenesis-related proteins, and structural and metabolic proteins. According to the database, some of the identified proteins have also been described as significant sensitizers. It is also known that *N*-linked glycans of plant glycoproteins are among the most abundant environmental immune determinants. In contrast, nonenzymatic glycosylation is most common in pathogenesis-related proteins, particularly α-amylase inhibitors, serpins, and LTP proteins.

**Table 3:** Summary of Essential Proteins and their PTMs Identified in Barley

				PTM		
protein classification/biological function	protein	UniProt entry	mass (Da)	glycation	glycosylation	phosphorylation
storage proteins/prolamins	D-hordein	Q40054	75 108		347 NPSG	
	γ-hordein	P17990	34 737		24 NPSV, 287 NCST	
	B1-hordein	P06470	33 422	K144(Hex1)		
	γ-hordein-3	P80198	33 189	K155(Hex1)	9 NPSG	
	B3-hordein	P06471	30 195	K115(Hex2)		
	putative γ2-hordein	Q70IB4	29 033	K242(Hex1)	4 NPSV, 237 NCST	
	C-hordein	P80198	12 180		24 NPSS	
pathogenesis-related proteins (PRs)/protective–stress response proteins	serine protease inhibitor serpin-Z4	P06293	43 276	K276(Hex1), R379(Hex1)	92 NESS, 170 NTTK	
	serpin-Z7	Q43492	42 821	R377(Hex1)	154 NVTA, 171 NTTR, 205 NGST, 316 NLSE	
	peroxidase 1	P27337	32 976		158 NSSR, 265 NDTT	
	basic pathogenesis-related protein PR5	O23997	25 172		187 NYSM	
	germin-like protein	Q43487	24 692		77 NVTL	
	barperm 1	O22462	21 656		167 NYSK	
	α-amylase/trypsin inhibitor CMd	P11643	18 526	K83(Hex2)		
	cold-regulated protein	Q9FSI8	17 613	K140(Hex1)		
	α-amylase inhibitor BDAI-1	P13691	16 429	K44(Hex2)	78 NISN	
	glycine-rich RNA-binding protein	Q43472	15 926			S87-p
	α-amylase inhibitor BMAI-1	P16968	15 816		125 NGTD	
	α-amylase/trypsin inhibitor CM16	P16159	15 782		124 NLTT	
	α-amylase/trypsin inhibitor CMa	P28041	15 500	K121(Hex1)		
	thionin BTH7	Q42838	14 676	K61(Hex2)		
	dehydrin DHN1	P12951	14 239	K13(Hex1)		
	nonspecific lipid-transfer protein 1	P07597	12 301	K35(Hex1), K78(Hex1)		
	nonspecific lipid-transfer protein 2	P20145	10 357	K75(Hex1)		
	protease inhibitor 13	M0XBS5	8 963	K64(Hex2)		
structural and metabolic proteins	α-mannosidase	F2DJN8	112 905		30 NTSA, 58 NNSI, 273 NVTR, 465 NITY, 475 NFSQ, 526 NASS, 727 NKTF	
	glutamate receptor	F2EAV8	100 825		377 NFTG, 416 NYSG, 485 NNTG, 518 NGSQ, 522 NPSY, 549 NRTR	S888-p
	ubiquitin-like protein	F2E698	94 306	K687(Hex2)		
	potassium transporter HAK2	Q9M7K3	86 015			S652-p
	β-d-xylosidase	Q8W011	83 529		203 NSSD, 432 NASL, 473 NVSN, 710 NATD	
	inorganic pyrophosphatase	Q06572	79 533			S42-p, S119-p
	hexose transporter, HvSTP	Q8GT52	79 341			S382-p, S363-p
	calcium-dependent protein kinase 2	Q06850	68 254			S314-p
	heat shock protein 70	Q40058	67 016			S45-p, T95-p, Y44-p
	purple acid phosphatase isoform a	C4PKL2	60 298		140 NYTS, 205 NTTS, 236 NGTG, 292 NKTF, 414 NYTL, 465 NFTS, 500 NETH, 536 NSTR	T186-p
	β-amylase	Q9FUK6	59 312	K291(Hex1)	235 NDTP, 247 NGTY, 336 NFTC, 400 NQSG	
	sodium/proton exchanger, HvNHX1	Q852S3	59 246		52 NESI, 295 NVTE, 370 NLTK	S457-p, S461-p
	β-glucosidase	Q40025	57 445		86 NGTA, 356 NQTP, 408 NPTM, 423 NVSI	
	chaperonin HSP60	F2DVX3	56 162		64 NATN, 67 NDTA, 124 NTSE, 386 NATK	T35-p
	serine carboxypeptidase II	P08818	52 625		148 NTSS, 159 NRTA, 291 NISS, 341 NVTG, 347 NYTW, 352 NCSD, 472 NVTV	
	β-tubulin	P93176	50 194		184 NATL, 337 NSSY, 370 NSTS	S172-p
	malate dehydrogenase	F2D4W6	37 357		20 NPTR, 167 NSTV	S63-p, S196-p
	glyceraldehyde-3-phosphate dehydrogenase	P26517	36 514			T68-p
	aldose reductase	P23901	35 807		167 NYTV, 238 NKTP, 303 NKTH	
	gluthation -S-transferase	P30111	32 580			S116-p
	14-3-3-like protein A	P29305	29 352			S239-p
	ribose-5-phosphate isomerase 2	Q9AYT7	28 669		106 NITD	S107-p, S194-p
	26 kDa endochitinase 2	P23951	28 156	K132(Hex1)		
	triosephosphate isomerase	F2EHF8	26 779			S192-p
	2-Cys peroxiredoxin BAS1, chloroplastic	Q96468	23 299			S106-p, S127-p
	calmodulin	P62162	16 832			S54-p, S82-p, T80-p
	nucleoside diphosphate kinase	F2CXV3	16 646			S118-p, T92-p, T5-p

### 4.4 Alternative Modifications

Thanks to rapid advances in high-throughput mass spectrometry, it is now possible to comprehensively detect and quantify PTM sites within a single proteomics experiment, including phosphorylation and glycosylation, as well as ubiquitination, SUMOylation, acetylation, and succinylation. Succinylation, a type of PTM occurring on lysine residues, has been shown to play an important role in gene transcription and plant growth. Wang et al. recently suggested that protein phosphorylation and succinylation are often occurring PTMs that may play a key regulatory role in the response of barley roots to phosphate stress. This finding is interesting given that phosphate stress represents an important environmental factor limiting plant growth and development.^89^

Because the function of small RHO-type G-proteins as signaling centers and key regulators of cell polarity must be strictly controlled, PTMs of RHO proteins, such as lipid modifications, phosphorylation, and ubiquitination, have recently been described. It was found that the ubiquitination site is maintained in all barley ROPs. This indicates that the lysine residue belonging to RACB K167 is a general target for ubiquitination and that this regulates the amount of protein.^90^

SUMOylation is a key player in plant immunity and, thus, plays a positive role in fungal resistance. By studying the molecules accumulated in barley, a certain level of resistance to *Fusarium graminearum* was confirmed, leading to the identification of resveratrol, a candidate inhibitor of SUMO proteases.^91^

Our brief review concerning the PTMs of barley was mainly focused on the study of individual PTMs. Since studies of the interactions of PTMs in plants have only recently emerged, it will undoubtedly be desirable to focus attention on this area in the future.

## 5 Other Important Crops

As mentioned above, several efficient methods have been developed for *N*-glycoprotein enrichment according to different enrichment mechanisms. These methods were applied to the glycoproteomic analysis of various plant species, including *Arabidopsis* leaf cell walls, *Brachypodium distachyon* seedling leaves, ripe tomato fruits, maize seeds, and seedling leaves and aleurone layers of barley, wheat, and rice. Since wheat (*Triticum aestivum* L.) is a globally important cereal crop and PTMs of proteins are widely involved in the regulation of plant abiotic stress, Wang et al. performed the first analysis of the *N*-glycoproteome of wheat seedling leaves by enrichment glycosylation using the HILIC method and tandem mass spectrometric analysis with the use of an Orbitrap Q Exactive Plus hybrid quadrupole-Orbitrap mass spectrometer. The results of their study showed that glycosylation sites throughout the cell are more likely to be located on a random coil, and the study also showed that glycosylation modification maintains the stability of the protein structure.^92^ Because drought has a severe limiting effect on wheat growth and yield formation, a combination of a proteomics approach and Pro-Q Diamond gel staining was used to determine that several proteins are phosphorylated and up-regulated under drought conditions. Luo et al. identified 58 wheat proteins that were phosphorylated among 112 differentially accumulated proteins in response to the water deficit. Significantly, phosphorylation of heat shock proteins, for example, was specifically induced by drought stress, which may suggest an important role in drought resistance.^93^ Rice (*Oryza sativa L*.) is also one of the most frequently consumed cereals. Recently, proteomic technology has been used to determine differentially expressed rice proteins related to starch biosynthesis and to identify PTMs that target starch biosynthesis proteins. Most proteins related to starch biosynthesis are essentially elevated at 6–20 days after flowering and decrease under conditions of high temperature. Some of these have been identified as being targeted by phosphorylation, lysine acetylation, succinylation, 2-hydroxyisobutyrylation of lysine, and malonylation. Phosphoglucomutase, an important enzyme that transfers the phosphate group on the α-d-glucose monomer from position 1 to position 6, is commonly the target of five types of PTMs.^94^ In the endosperm of maize (*Zea mays L.*), starch synthesis (SSI, SSIIa) and starch branching (SBEIIb) enzymes form a trimeric complex, with SBEIIb being phosphorylated.^95^ The formation of the complex is triggered by ATP and degrades alkaline phosphatase. These findings confirm the hypothesis that phosphorylation-dependent heteromeric enzyme complex formation facilitates amylopectin formation in starch granules.^96^

Another important task for cereals is the detection of allergenic proteins. Allergen characterization has recently been addressed by allergenomics, which can be used to identify putative allergens over a short time. Allergens present in cereal grains involve proteins belonging to the groups of albumins, globulins, prolamins, and glutenins.^97^ Among the important allergens in wheat and barley flour is the α-amylase/trypsin inhibitor group, most of which are glycated or glycosylated.^98^ Barley LTP1 and protein Z4 have also been confirmed to be significant beer allergens.^99^

## 6 Conclusion

The works discussed in this review report different techniques used to identify barley PTMs. Advances in sample preparation, mass spectrometry, and bioinformatics are enabling more accurate and faster identification, quantification, and characterization of proteins as well as their modifications.

Proteomic methods based on LC-MS/MS are currently available for the detection of different proteins and their PTMs due to their accuracy, precision, and sensitivity and robust quantitative capability. One of the most recent approaches uses Data Independent Acquisition (DIA)/Sequential Window Acquisition of All Theoretical Mass Spectra (SWATH) LC-MS/MS with bioinformatics workflows to identify and measure PTMs in order to investigate the underlying protein biochemistry of various samples. Extensive complexity and diversity of PTMs were found in the proteomes, particularly proteolysis from barley proteases, O-glycosylation of secreted yeast glycoproteins, and the glycation of barley proteins with malto-oligosaccharides.

Another method is the electrochemical analysis of glycoprotein samples by combining a microfluidic device with voltammetric analysis on amalgam electrodes. Glycoproteins are electroactive on electrodes containing carbon and mercury. Their intrinsic signals obtained by square wave voltammetry or chronopotentiometric constant current sampling are very high with an effective baseline correction. At the same time, they are more developed, and much lower protein concentrations can be measured compared to linear sweep voltammetry. The literature shows that the combination of electrochemical methods with microfluidic devices is sporadically used. However, it should be mentioned that electrochemical methods offer accurate, simple, and inexpensive analysis.

As all knowledge of the proteins involved, their biological role, structure, and function in food raw materials and final food products is essential for food processing, new low-cost, efficient, and robust analytical methods with higher sensitivity need to be developed.
